# Sensory neuronal P2RX4 receptors controls BDNF signaling in inflammatory pain

**DOI:** 10.1038/s41598-018-19301-5

**Published:** 2018-01-17

**Authors:** Sarah Lalisse, Jennifer Hua, Manon Lenoir, Nathalie Linck, François Rassendren, Lauriane Ulmann

**Affiliations:** 1 Institute of Functional Genomics, Université de Montpellier, Unité Mixte de Recherche, 5302 CNRS, 141 rue de la cardonille, F34094 Montpellier, France; 2 Unité de recherche U1191, INSERM, Montpellier, France; 3 LabEx Ion Channel Science and Therapeutics, 141 rue de la cardonille, Montpellier, F34094 France

## Abstract

Chronic inflammatory and neuropathic pains are major public health concerns. Potential therapeutic targets include the ATP-gated purinergic receptors (P2RX) that contribute to these pathological types of pain in several different cell types. The purinergic receptors P2RX2 and P2RX3 are expressed by a specific subset of dorsal root ganglion neurons and directly shape pain processing by primary afferents. In contrast the P2RX4 and P2RX7 are mostly expressed in myeloid cells, where activation of these receptors triggers the release of various pro-inflammatory molecules. Here, we demonstrate that P2RX4 also controls calcium influx in mouse dorsal root ganglion neurons. P2RX4 is up-regulated in pain-processing neurons during long lasting peripheral inflammation and it co-localizes with Brain-Derived Neurotrophic Factor (BDNF). In the dorsal horn of the spinal cord, BDNF-dependent signaling pathways, phosphorylation of Erk1/2 and of the GluN1 subunit as well as the down regulation of the co-transporter KCC2, which are triggered by peripheral inflammation are impaired in P2RX4-deficient mice. Our results suggest that P2RX4, expressed by sensory neurons, controls neuronal BDNF release that contributes to hyper-excitability during chronic inflammatory pain and establish P2RX4 in sensory neurons as a new potential therapeutic target to treat hyperexcitability during chronic inflammatory pain.

## Introduction

Pain is a sensory modality that is encoded by specialized nociceptive neurons in the dorsal root ganglion (DRG). Nociceptive neurons express specific markers and are small or intermediate in diameter compared to the larger proprioceptive neurons. Nociception is an adaptive phenomenon that protects the body from insults, whereas chronic pain resulting from various pathological conditions, is triggered by non-noxious stimuli and is harmful rather than useful. Therefore attacking pathological pain is an intense area of medical research.

Among different signaling pathways involved in pain processing, extracellular ATP has emerged as a key molecule, that activates different membrane receptors, especially the ATP-gated (P2RX) channels and the metabotropic G-protein coupled P2Y receptors^1^.

There are seven P2RX subunit genes (P2RX1-7) whose encoded proteins form trimeric homo- or heteromeric channels. All the P2RX subunit mRNAs are expressed in DRG neurons^2^, but evidence for their functional expression and potential involvement in pain processing remains limited to a few of them^3,4^. P2RX3 and P2RX2/3 receptors are mainly expressed in small-to-medium diameter C- and Aδ-nociceptive neurons, and are localized on both peripheral and central termini of the fibers^5^.

Studies using different pain models and approaches provided compelling evidence that P2RX3 is implicated in chronic pathological pain. Particularly, selective P2RX3 antagonists, antisense treatment or genetic inactivation significantly reduce inflammatory and neuropathic pain behaviors in mice^6^ indicating that these receptors contribute to pain processing^7^. Gene inactivation of *p2rx4* and *p2rx7*^8–10^ as well as highly potent specific P2RX7 antagonists^11^ clearly implicated P2RX4 and P2RX7 in inflammatory and neuropathic pain also, although the mechanisms underlying their respective contributions are not fully understood. While P2RX7 is predominantly found in myeloid cells, P2RX4 is much more widespread and it is expressed in both macrophages and neurons. Sensory neurons mostly express P2RX4 mRNA^12^, and data obtained from deep RNA sequencing of individual DRG neurons clearly confirmed the presence of P2RX4 mRNA in distinct populations of DRG neurons, including nociceptive neurons^13^. However, direct evidence for a function for P2RX4 in sensory neurons is still lacking.

Brain-Derived Neurotrophic Factor (BDNF) is expressed in neurons and in immune cells. It has multiple roles including a neurotrophic factor during development and a role in adult learning^14^. BDNF is also involved in neuropathic pain: A series of studies has demonstrated that *de novo* P2RX4 expression triggers BDNF release from activated spinal microglia, which are the resident macrophages of the central nervous system. BDNF binding to neuronal TrkB receptors down regulates the cotransporter KCC2, which causes a switch from inhibitory to excitatory GABA transmission, then underlying allodynia and mechanical hypersensitivity^9,15,16^. P2RX4 is thus an enigmatic but interesting potential therapeutic target to treat pathological pain and indeed a P2RX4 antagonist was recently developed in a model of herpetic pain^17^.

As during neuropathic pain, P2RX4-deficient mice do not develop mechanical hypersensitivity in a model of acute inflammatory pain. In this condition, in wild-type mice, P2RX4 in skin-resident macrophages is likely to contribute to prostaglandin E2 release and to the subsequent sensitization of sensory peripheral nerve endings^18^. Although P2XR4^−/−^ mice do not develop hypersensitivity during chronic inflammatory pain, the mechanisms underlying P2RX4’s contribution to this lack of pain remain poorly characterized. In particular, it is not known whether or not P2RX4 in sensory neurons controls chronic inflammatory pain processing.

Here we show that P2RX4 is expressed by sensory neurons and upregulated under conditions of long lasting inflammation. We also find that neuronal P2RX4 mediates BDNF signaling in the dorsal horn of the spinal cord during long lasting inflammation.

## Results

### P2RX4 is expressed in sensory neurons

**Figure 1:**
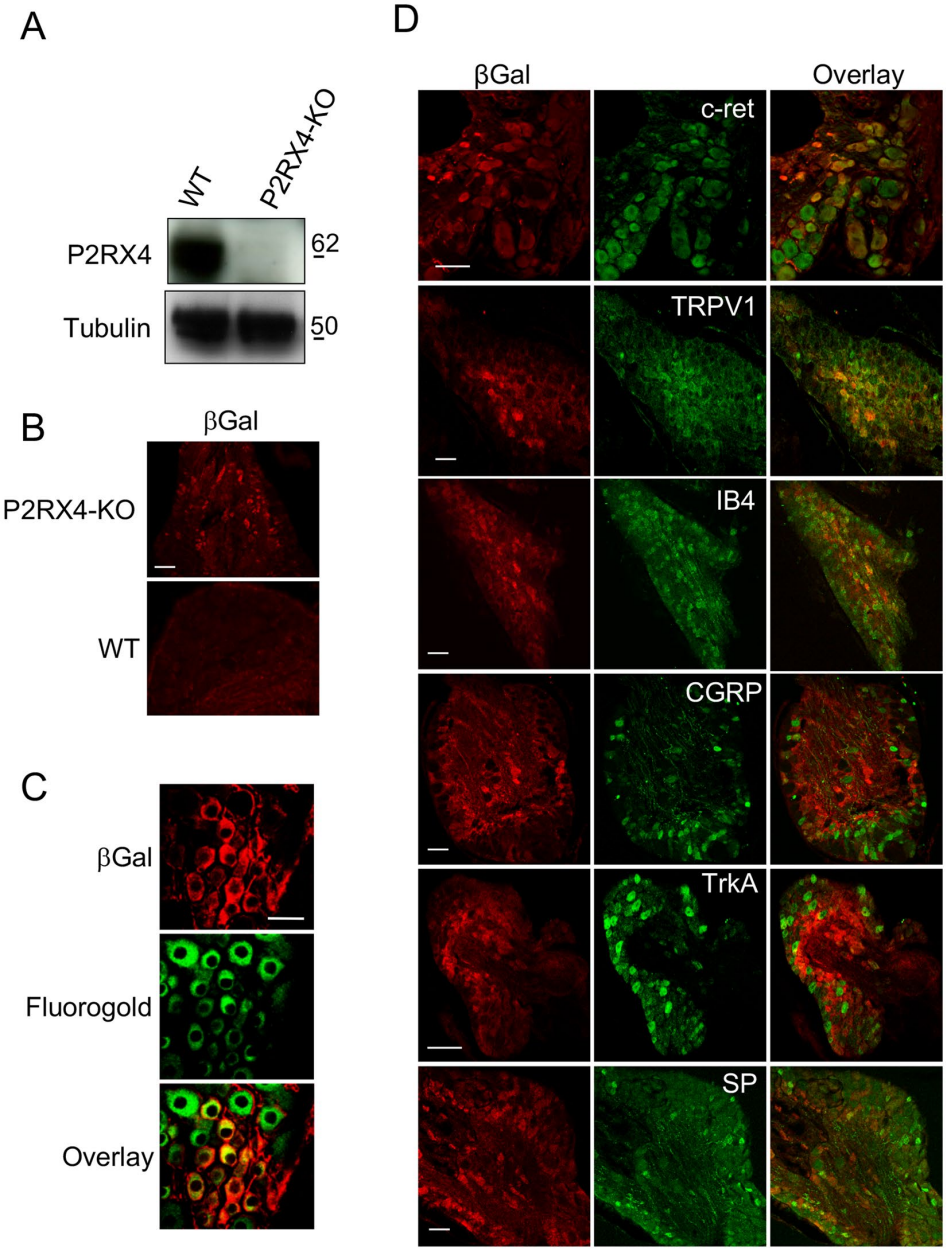
P2RX4 expression in Dorsal Root Ganglion neurons. (**A**) Representative cropped western blotting analysis of lumbar DRG extract indicates the presence of a 60 kDa band in P2RX4^+/+^ DRG that is absent in extracts from P2RX4^−/−^ DRG. N = 3 independent experiments, n = 3 mice per genotype. (**B**) β-galactosidase staining was used as a surrogate marker for P2RX4 in DRG sections. Only P2RX4^−/−^ neurons are immuno-positive for ßgal compared to sections from P2RX4^+/+^ DRG. Scale bar 100 µm. (**C**) DRG neurons that express ß-gal innervate paw skin. Fluoro-Gold B, a retrograde marker of neurons was injected in paw-skin and L5/L6 DRG were removed for staining a week later. βgal immunostaining reveals a population of neurons that are labeled for both ßgal and Fluoro-Gold B. Scale bar 50 µm. (**D**) In nociceptive neurons, ßgal is mainly expressed in c-ret-, TRPV1- and IB4-positive neurons, but scarcely in others populations (CGRP, TrkA or Substance P (SP) expressing cells). Representative images of DRG sections stained with βgal and makers of nociceptive neurons. Scale bars 50 µm.

The expression of P2RX2 and P2RX3 in sensory neurons is well established and they are clearly involved in the transmission of inflammatory pain. Evidence for expression of P2RX4 in dorsal root ganglion neurons is lacking, while P2RX4-deficient mice do not develop hypersensitivity during long-lasting inflammation^18^. To determine whether P2RX4 might be implicated in neuronal sensory transmission, we used western blotting and immunohistochemistry on mouse Dorsal Root Ganglia (DRG) to investigate P2RX4 expression. A 60 kDa band was clearly detectable in protein extracts from wild-type DRG (Fig. 1A). We next analyzed the cellular localization of P2RX4 in DRG by immunohistochemistry. Because none of the P2RX4 antibodies we tested work on DRG sections, we took advantage of the fact that the P2RX4 knockout mouse was created by replacing the P2RX4 gene with an *E coli*. β-galactosidase LacZ gene (βgal). Staining of DRG from P2RX4^−/−^ mice with anti-βgalactosidase antibody can thus give an indication of the likely intended whereabouts of and report on normal *p2rx4* transcriptional activity^19^. ßgal immunostaining of P2RX4^−/−^ DRG slices revealed a neuronal expression which was totally absent in wild-type as expected (Fig. 1B). ßgal immunostaining was also observed in non-neuronal cells (likely satellite cells) surrounding neuronal cell bodies.

We also determined whether neurons expressing P2RX4 emit peripheral axons to the skin that could involve P2RX4 in the detection of peripheral sensory stimuli. For this, we performed intra-plantar injection of the retrograde neuronal cell body marker Fluoro-Gold B to test for the presence of neuronal cell bodies in the DRGs that express P2RX4. We observed neurons that were positive for both Fluoro-Gold B and βgal indicating that P2RX4-positive neurons emit axonal processes toward their periphery (Fig. 1C).

Because previous studies, including ours, implicated P2RX4 in pain transmission^10,18^, we thus investigated whether P2XR4 was specifically present in nociceptive neurons by co-staining with the C and Aδ neuronal markers c-ret, TRPV1, IB4, CGRP, substance P and TrkA receptors. The proxy for P2RX4, βgal, mainly co-localized in c-ret, TRPV1 and IB4-positive neurons, which implies that P2RX4 is expressed in nociceptive neurons (Fig. 1D).

### Sensory neuronal P2RX4 participates in calcium signaling

**Figure 2:**
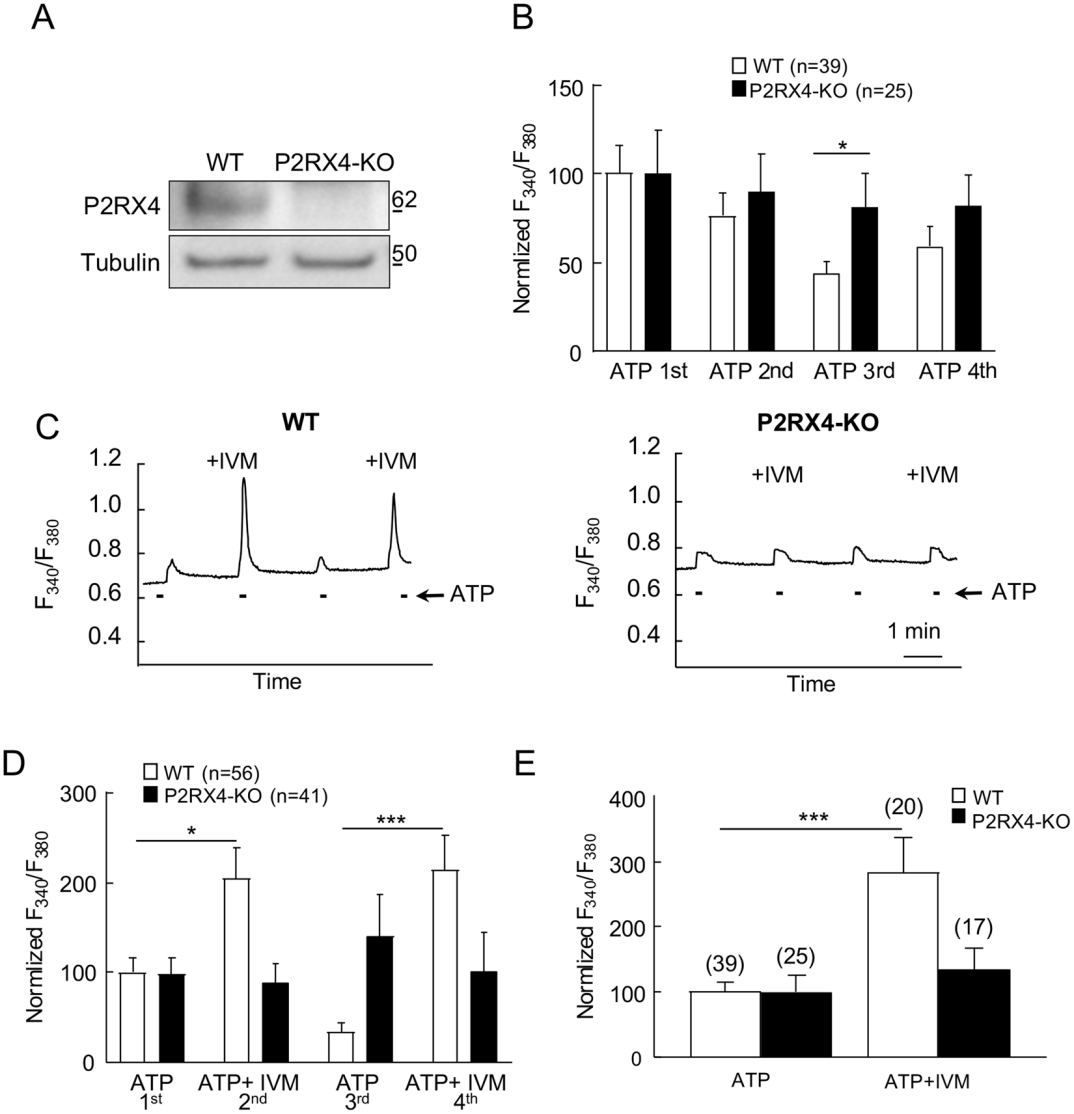
P2RX4 evokes calcium signaling in cultured DRG neurons. (**A**) Representative cropped western blot analysis of P2RX4 expression in DRG neurons in primary culture. N = 2 independent cultures. (**B**) Analysis of ATP-evoked calcium signals in cultured DRG neurons. Quantitative analysis of the Fura-2 fluorescence ratio upon repetitive ATP applications (20 µM) in neurons from WT and P2RX4-deficient mice. Data were normalized to the first ATP response. Upon repeated ATP applications, calcium signal in WT neurons decrease significantly, whereas ATP responses remained stable in P2RX4^−/−^ cells. N = 2 independent experiments. (**C**) Representative recordings of ATP− and ATP+ ivermectin (IVM)-evoked calcium increase in single neurons from P2RX4^+/+^ and P2RX4^−/−^ mice. ATP-evoked calcium signal is potentiated by IVM (3 µM) only in P2RX4^+/+^ neurons. (**D**) Population analysis of ATP-evoked fluorescence signals from (**C**), normalized to the first ATP response. IVM significantly increases ATP response in P2RX4^+/+^ neurons but does no effect in P2RX4^−/−^ cells. Note that IVM reverses the decrease of calcium response evoked by repetitive ATP application. N = 3 independent experiments, n = 56 and 41 total cells, for WT and P2RX4^−/−^, respectively. (**E**) Analysis of calcium signals evoked by a single ATP or ATP and IVM application in WT and P2RX4^−/−^ naïve neurons. Results are normalized to signal evoked by ATP alone. IVM potentiates ATP-evoked signals only in WT mice. N = 2 independent experiments, the number of neurons are inside brackets. Results are expressed as mean ± SEM, One way ANOVA, *p < 0.05, ***p < 0.005.

We next used calcium imaging of primary cultures of DRG neurons to investigate whether P2RX4 is functional in sensory neurons. We first verified by western blotting that P2RX4 is expressed in primary DRG neuronal cultures (Fig. 2A). To test if P2RX4 is functional in neurons, we next compared ATP-evoked calcium signals in DRG neurons from WT and P2RX4-deficient mice. Calcium signals were evoked by repetitive stimulations with 20 µM ATP, for 10 s every 2 minutes. The initial ATP application evoked a similar increase of fluorescence in P2RX4^+/+^ and P2RX4^−/−^ neurons, however, upon repetitive applications, ATP-induced signal decreased significantly in neurons from WT compared to P2RX4-deficient mice, suggesting that repetitive ATP applications induces P2RX4 desensitization (Fig. 2B). We then used ivermectin (IVM), which is a positive allosteric modulator of P2RX4, to reverse P2RX4 desensitization in WT neurons^20^. ATP or ATP plus IVM (3 µM) were alternatively applied to neurons every two minutes. In the presence of IVM, ATP-evoked calcium signals were significantly increased in 38% of the recorded neurons from WT mice. However IVM had no effect on the ATP response of P2RX4-deficient neurons (Fig. 2C, D). Our results indicate that the first ATP application is similar between WT and P2RX4-KO neurons. To reveal the contribution of P2RX4 in this response, ATP was co-applied with IVM as soon as the first application. In this case, IVM increases the ATP response only in WT neurons when compared with ATP alone (Fig. 2E). These results clearly indicate that a subpopulation of DRG sensory neurons express functional P2RX4, which contributes to ATP-evoked calcium signals in condition of sustained ATP concentration as observed during peripheral inflammation.

### Peripheral inflammation upregulates P2RX4 in sensory neurons

**Figure 3:**
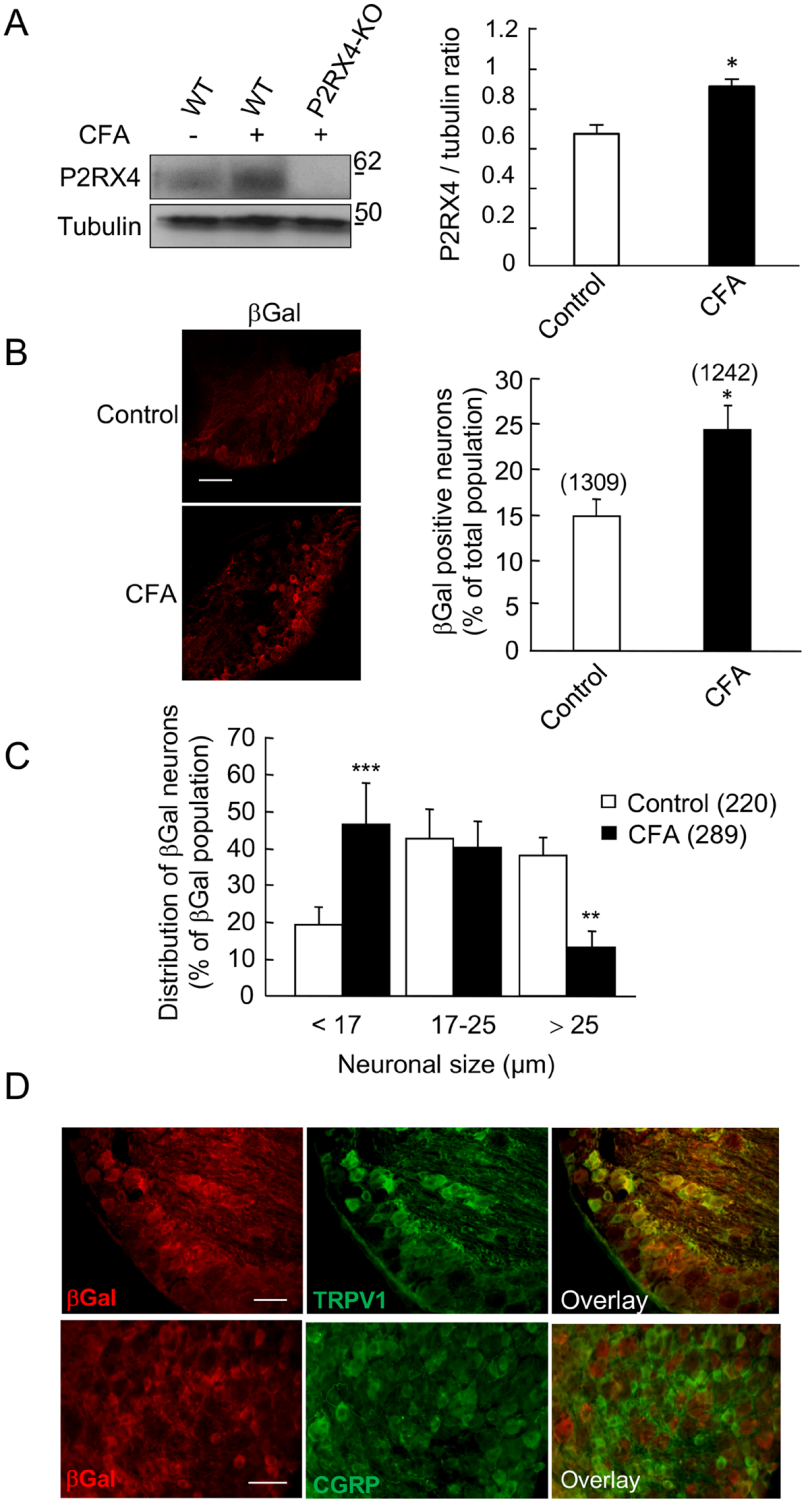
Peripheral inflammation up-regulates P2RX4 in DRG neurons. (**A**) Representative cropped western blot of P2RX4 expression in lumbar DRG extracts. 24 h following CFA injection in the hind paw an increase of P2RX4 expression is seen in extracts of ganglion, n = 3 mice per lane. Left panel, western blot quantification of the P2RX4 to tubulin ratio in ganglia. CFA injection induces a significant increase of P2RX4 expression in DRG. N = 3 independent experiments. Unpaired t-test, *p < 0.05. (**B**) Representative images of β-galactosidase (βgal) expression in a lumbar DRG section (left panel) and quantification of the proportion of the number of positive neurons among the total population (right panel), 24 h after CFA injection. A significant increase in the number of positive neurons was detected in inflammatory conditions. Scale bar 100 µm, unpaired-t-test, *p < 0.05. (**C**) Soma size of neurons distribution of P2RX4 positive neurons 24 h after CFA infection. The percent of small neurons labeled with βgal antibody is significantly increased, indicating an increase of P2RX4, mainly in small receptive pain neurons. χ^2^ analysis of contingency table, ** p < 0.01, ***p < 0.005. (**D**) Representative immuno-histochemistry of DRG sections showing that 24 h after CFA injection, βgal is upregulated in the same population of sensory neurons (TRPV1 positive and CGRP negative) as in control conditions (compare with data shown in Fig. 1). Scale bar 50 µm.

In pathological conditions, such as neuropathic pain or epilepsy, P2RX4 expression is up-regulated in both hippocampal and spinal neurons and microglia^9,21,22^. We thus analyzed whether a similar phenomenon could be observed in peripheral sensory neurons following a peripheral inflammatory challenge. We compared P2RX4 expression in basal conditions and 24 h after intra-plantar injection of Complete Freud Adjuvant (CFA), which induces long lasting inflammatory pain. Western blotting experiments show that CFA elicited a significant increase of P2RX4 expression in P2RX4^+/+^ DRGs (Fig. 3A) (0.68 ± 0.04 in control *vs* 0.94 ± 0.03 in CFA condition, N = 3 independent experiments, n = 3 mice per condition, unpaired Student’s *t*-test, p < 0.05). A similar increase of P2RX4 expression was observed in the central processes, suggesting that P2RX4 is up-regulated and sent towards the central axons (Sup. Fig. 1). Consistent with this finding, peripheral inflammation lead to an increase of the proportion of βgal positive neurons in P2RX4^−/−^ DRG compared to control condition (23.6 ± 2% *vs* 14.7 ± 1.9%, n = 1242 and 1309 cells, respectively, 5 animals per condition, unpaired Student’s t-test, p < 0.05) (Fig. 3B). Three categories of DRG neurons can be determined according to the soma size; Small and intermediate neurons, which are the C and Aδ nociceptive neurons, and the large mechanical neurons^23^. We used the size of the neuronal body to investigate whether the up-regulation of P2RX4 expression was specific to any subpopulation of neurons. Morphological analysis of βgal-positive neurons performed on lumbar P2RX4^−/−^ DRG sections revealed that P2RX4 is evenly expressed in the three populations in untreated control conditions (Fig. 3C). Compared to control conditions, CFA injection induced a significant increase of the proportion of the βgal-positive neurons among the small neurons (46.3 ± 11.5% *vs* 19.3 ± 4.8%, Ҡ^2^ squared test, CFA *vs* control, respectively). There was no change in the intermediate population and a decrease in βgal staining in large neurons. The total number of neurons and the distribution of the soma size were not altered by CFA (data nsot shown). In inflammatory conditions, the increase of βgal occurred in the same neuronal population as in control conditions (TRPV1 positive and CGRP negative neurons, Fig. 3D). These results indicate that during long lasting inflammation, P2RX4 is upregulated in small nociceptive neurons.

### Pain behavior in female P2RX4

Inflammatory pain behavior for the P2RX4-deficient mice has been previously reported only in males^10,18^. Yet recent studies have suggested a sex difference in the contribution of P2RX4 to neuropathic pain^24^. We investigated whether females P2RX4-deficient mice will present the same absence of mechanical hypersensitivity 24 hours following CFA injection in the hind paw as male do. As shown in Sup. Fig. 2, females P2RX4-deficient mice did not develop any hypersensitivity to CFA as compared to WT, suggesting that P2RX4 equally contributes to inflammatory pain in male and female mice.

### P2RX4 drives neuronal BDNF signaling

**Figure 4:**
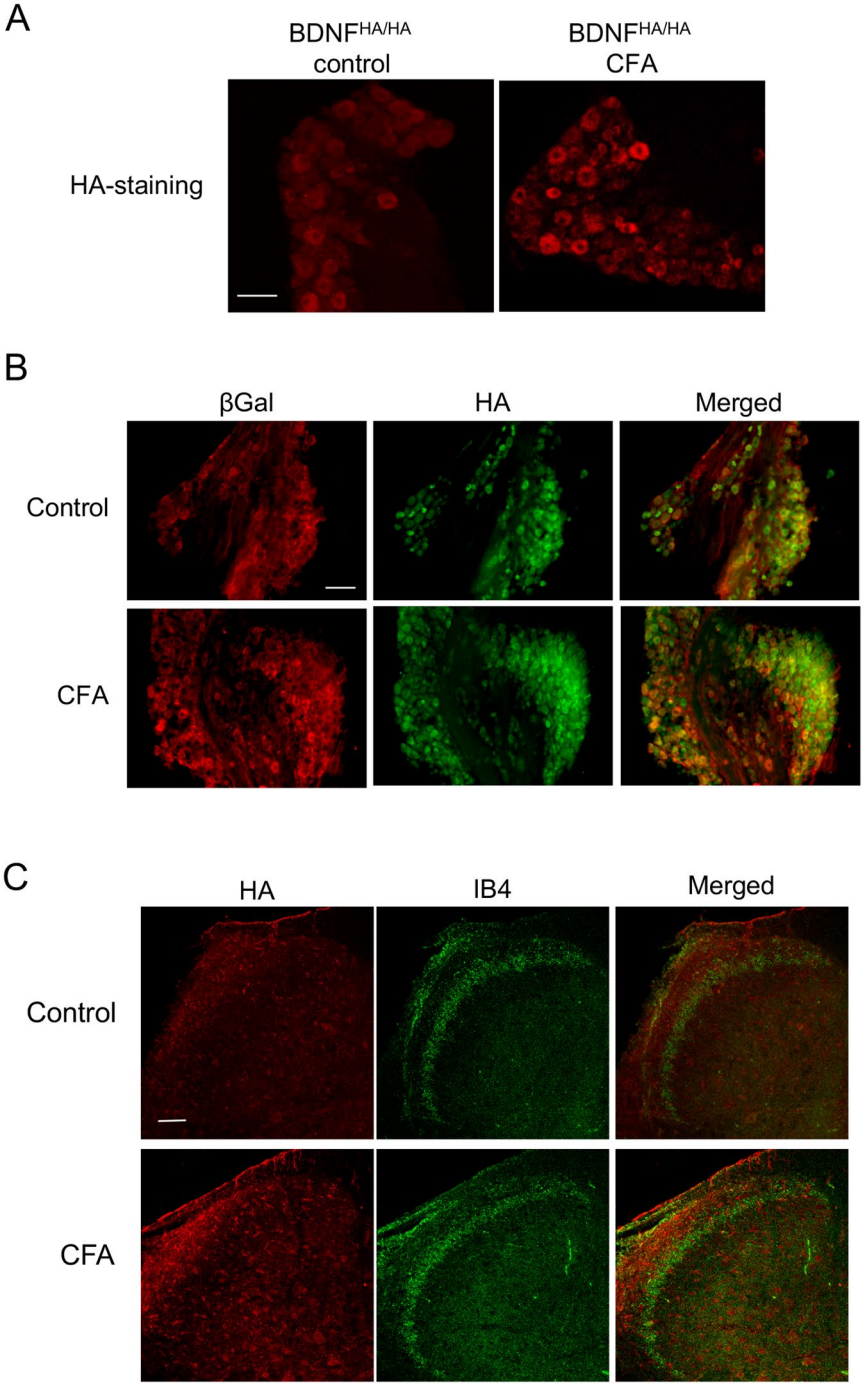
BDNF is upregulated in P2RX4 neurons 24 h after peripheral inflammation. BDNF expression was analyzed in BDNF-HA mice in which a HA epitope is added at the carboxyl terminus of BDNF. (**A**) In DRG sections of BDNF^HA/HA^ mice, HA-immunofluorescence was detected in control conditions (left panel) and the number of labeled neurons increases 24 h after CFA injection (right panel). Scale bar 100 µm. (**B**) Co-immunostaining of βgal and BDNF in control and 24 h after CFA injection in DRG sections. βgal is expressed in a subset of BDNF-positive neurons. Scale bar 50 µm. (**C**) Representative pictures of Isolectin B4 (IB4) and HA staining in the spinal cord in control and CFA-conditions. Note the increase of BDNF staining in lamina I in CFA conditions and the absence of co-localization of the two markers. Scale bar 50 µm.

In neuropathic pain, P2RX4 is thought to mediate BDNF release from spinal microglia, thereby eliciting tactile allodynia^25^. We therefore hypothesized that under inflammatory conditions, neuronal P2RX4 might control the release of BDNF from central termini of nociceptive neurons to the dorsal horn of the spinal cord. To unambiguously detect BDNF, we used BDNF^HA/HA^ knock-in mice, in which a HA tag is inserted at the carboxyl terminus of the BDNF coding sequence^26^. To confirm the specificity of the antibody, HA immunostaining reveals BDNF expression in CA3 hippocampal neurons in BDNF^HA/HA^ mice as previously described (Sup. Fig. 3). In DRG sections we observed a basal expression of neuronal BDNF-HA, and peripheral CFA injection strongly increased BDNF expression in DRG neurons (Fig. 4A). Having verified our experimental set-up, we analyzed whether BDNF and P2RX4 co-localized in DRG neurons, as this would be required for P2RX4 to control BDNF release. 24 h after CFA injection, BDNF-HA and βgal immunostaining indeed co-localized in small and intermediate size sensory neurons (Fig. 4B). Similarly, in the dorsal horn of the spinal cord, BDNF-HA staining increased in lamina I and co-localized with isolectin B4 in lamina II, as previously shown for BDNF immune staining in mice^27^ (Fig. 4C). To determine whether in condition of long-lasting inflammation sensory neurons are the principal cells releasing BDNF in the dorsal horn, we investigated whether paw injection of CFA could trigger microglial activation, and whether spinal microglia express BDNF. There was no visible change in the morphology or the number of microglia observed with Iba1 staining in the dorsal horn between control and CFA conditions as assessed by quantification of total surface occupied by microglia (Sup. Fig. 4A, B). Finally, double immunohistochemistry with Iba1 and HA antibodies showed that microglia did not express BDNF in inflammatory conditions (Sup. Fig. 4C), although some rare microglial cells seem to be positive to HA. Our results suggest that in long lasting inflammatory pain, spinal microglia do not release BDNF, and that BDNF is mostly release by sensory neurons termini as previously proposed^28^.

**Figure 5:**
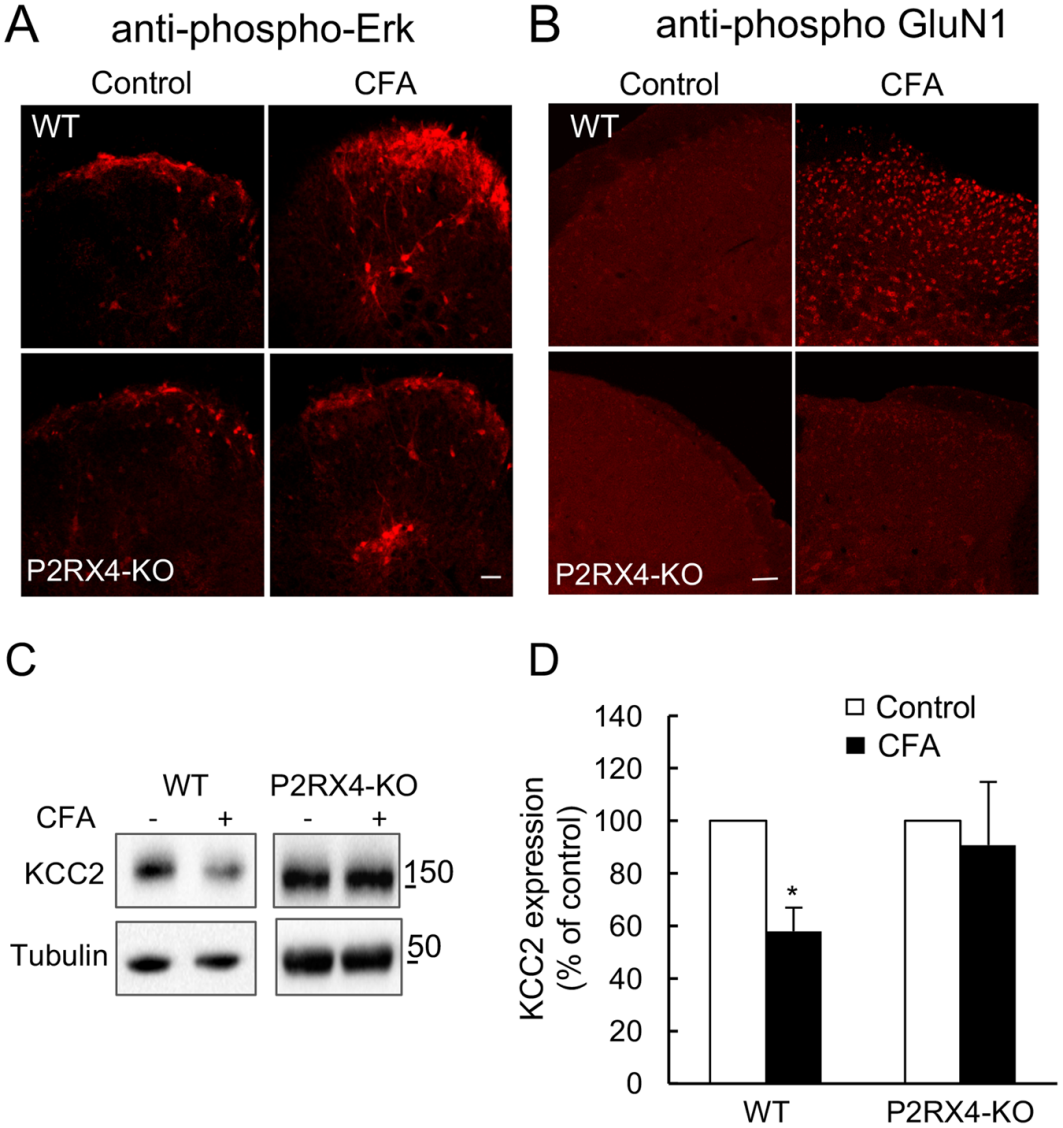
P2RX4 controls the spinal BDNF pathway under long lasting peripheral inflammatory conditions. Immunostaining analyses of the phosphorylation of the BDNF pathway proteins, Erk1/2 (**A**) and the GluN1 subunit of the NMDA receptor (**B**), in inflammatory conditions. Increased immunostaining of both phospho-Erk1/2 and phospho-GluN1 is observed in the dorsal horn, ipsilateral of the CFA injection in P2RX4^+/+^ mice, but it is absent in P2RX4^−/−^ mice. Note that, in P2RX4-deficient mice, phosphorylation of Erk1/2 is maintained in deep lamina. Scale bar 200 µm. (**C**) Representative cropped western blot of KCC2 expression in the dorsal horn of the spinal cord in control and inflammatory condition, ipsilateral of the CFA injection. (**D**) Quantification of KCC2 expression observed in (**C**). KCC2 is down regulated 24 h after CFA injection in the dorsal horn of WT mice, but not in P2RX4^−/−^ mice. N = 3 independent experiments, n = 2 mice per condition. One Way ANOVA, *p < 0.05.

In inflammatory conditions, BDNF is released by the primary afferent termini in the dorsal horn of the spinal cord where it contributes to mechanical hypersensitivity^29–31^. To explore the involvement of P2RX4 sensory neurons in BDNF release, we analyzed the specific signaling pathways that were previously described to be associated to BDNF release in the spinal cord^32^. We first investigated the Erk pathway, which is activated when BDNF binds to TrkB receptor. In WT mice, CFA-evoked inflammation triggered a strong induction of Erk1/2 phosphorylation in neurons from external lamina as well as in neurons from deeper lamina (Fig. 5A). In P2RX4-deficient mice, phospho-Erk1/2 was reduced in lamina I neurons but not altered in deeper lamina of the spinal cord. We next analyzed phosphorylation of the GluN1 subunit of the NMDA receptor, as it is a well-known target of the BDNF-TrkB pathway. As for Erk1/2, CFA induced a strong increase of the phospho-GluN1 immunostaining in dorsal horn neurons of WT mice that was absent in P2RX4-deficient animals (Fig. 5B).

BDNF is known to induce a down-regulation of the chloride/potassium co-transporter KCC2 in brain or spinal cord neurons in different pathological chronic conditions^33,34^. To confirm that neuronal P2RX4 conducts neuronal BDNF signaling in CFA conditions, we investigated the expression of KCC2 by western blot in the dorsal horn of CFA-injected WT and P2RX4^−/−^ mice. Compared to untreated mice, CFA injection downregulated KCC2 in the dorsal horn of the spinal cord in WT mice, but not in P2RX4-deficient mice (Fig. 5C, D). Overall, these results imply that neuronal BDNF release is dependent of P2RX4 in conditions of long lasting inflammation.

## Discussion

**Figure 6:**
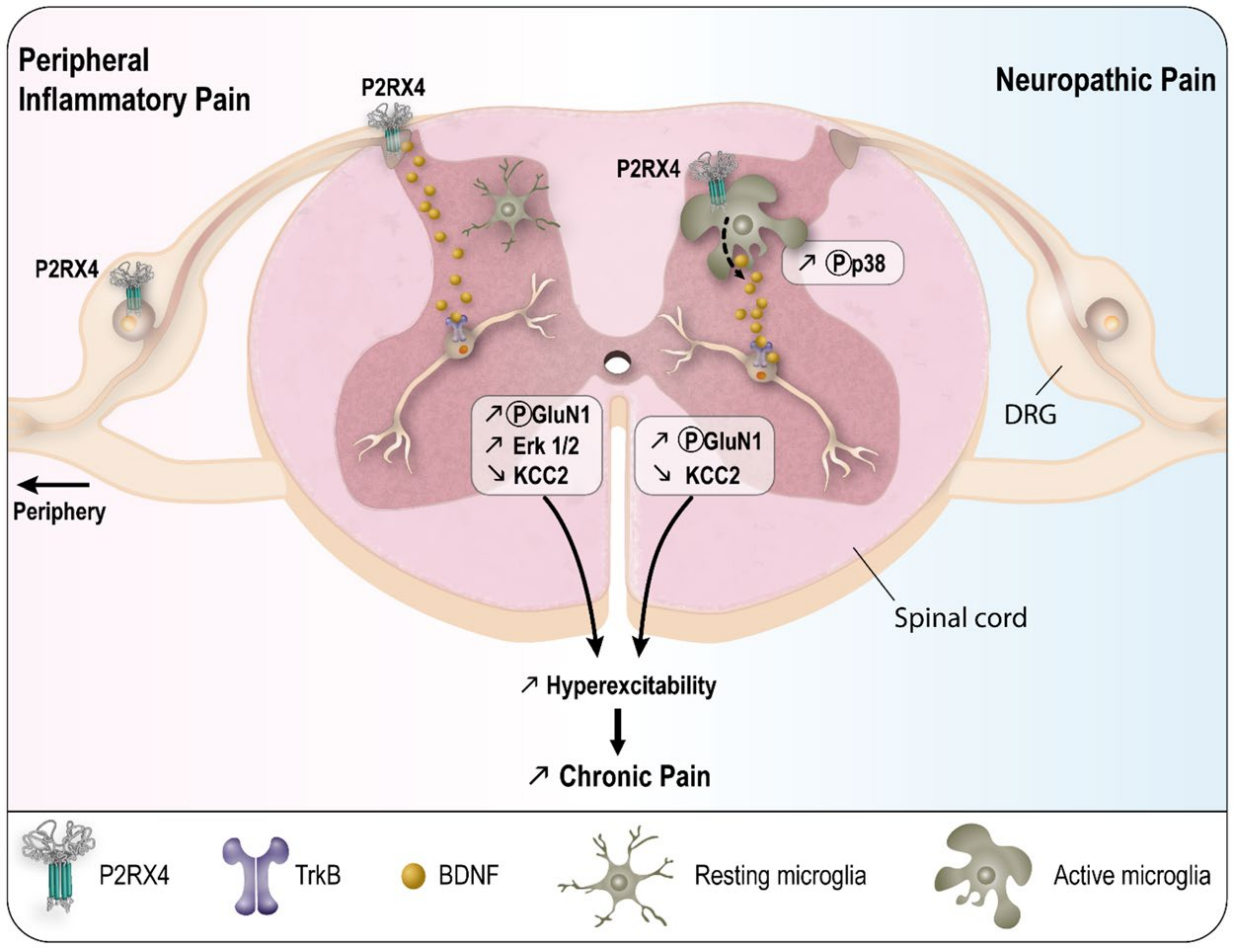
Summary of P2RX4 roles in long lasting inflammation and neuropathic pain. In condition of inflammatory pain, neuronal P2RX4 drives neuronal BDNF released in the spinal cord. During neuropathic pain, P2RX4 expressed by activated microglia is involved in microglial BDNF release. Both mechanisms induce a decrease of KCC2 and the development of chronic pain.

Several studies have demonstrated the presence of P2RX4 in different neuronal populations in hippocampus, cerebellum and cortex. In hippocampal pyramidal neurons, P2RX4 activation is triggered by episodes of high electrical activity (*e.g*. tetanus stimuli) and contributes to synaptic plasticity^19^. Our data support a specific role of P2RX4 expressed in sensory neurons and we also propose a new role of neuronal P2RX4 in a pathological condition, namely that of controlling the release of BDNF from sensory neurons to the spinal cord (Fig. 6).

Our immunohistochemistry data, using the detection of ß-galactosidase as a surrogate marker of P2RX4 expression, show that all types of sensory neurons express P2RX4. The half-life and subcellular localization of the ß-galactosidase marker certainly differ from that of P2RX4 and consequently its detection by immunostaining may be more sensitive compared to that of the P2RX4 protein. However, we found a good correlation between the up-regulation of P2RX4 expression, assessed by western blotting, and that of ß-galactosidase staining, in DRG, 24 hours after induction of peripheral inflammation. In addition, the half-life of βgal in sensory neurons is estimated at between 24–48 h^35^, which is close to that of recombinant P2RX4 expressed in HEK cells (data not shown). We feel therefore that βgal staining accurately reports on P2RX4 expression in our model. We cannot exclude that P2RX4 is also expressed in satellite cells surrounding neurons. However, our observation that P2RX4 is present in protein extracts from central axons, which are devoid of satellite cells, indicates that P2RX4 is expressed by neurons and could traffic to central termini. Our findings thus confirm for the first time previous *p2rx4* analysis showing the expression of the gene in distinct populations of DRG neurons^13^.

Our results strongly support the functional expression of P2RX4 in DRG neurons. In primary cultures of DRG neurons, recordings of ATP-evoked calcium signal further indicate a specific expression of P2RX4. Indeed, a positive allosteric modulator of P2RX4, IVM, enhanced ATP-evoked calcium signals in 38% of ATP-responsive cells, while it has no effect in neurons from P2RX4-deficient mice. In the absence of specific P2RX4 antagonist, evaluating the contribution of P2RX4 to ATP-evoked calcium signals is challenging. Our results show that upon repetitive agonist applications, ATP-evoked calcium signals significantly decrease in WT but not in P2RX4-deficient neurons. This decrease can be reversed by ivermectin, supporting that the reduction of ATP-evoked calcium signals reflects P2RX4 internalization. The P2RX4 transmembrane receptor is strongly permeable to calcium^36^. Therefore our results indicate that P2RX4 participates broadly in calcium signaling in sensory neurons and activate numerous intracellular pathways involved in sensory transmission. In resting conditions, P2RX4 is expressed in a subset of nociceptive neurons and this increases by a factor of two when peripheral inflammation is induced. A possible inference from our data is that the increase of P2RX4 expression is required to increase calcium signaling to modulate gene expression, to increase release of neurotransmitters^37^, or to increase sensory neuron excitability, which is a function that is currently solely attributed to the P2RX2 and P2RX3 receptors during pain processing.

We demonstrate that a population of sensory neurons co-express P2RX4 and BDNF in inflammatory conditions. Peripheral inflammation is known to induce a significant increase in BDNF synthesis in sensory neurons and it is anterogradely transported to the central termini to be released in the spinal cord in a neuronal activity-dependent manner^38,39^. In the light of our data it is conceivable that DRG presynaptic P2RX4-calcium activation causes primary sensory terminal release of BDNF in the dorsal horn of the spinal cord during long-lasting inflammation. This hypothesis is supported by the observation that known BDNF-dependent signaling pathways, in the dorsal horn of the spinal cord, are impaired in P2RX4-deficient mice. The affected pathways include phosphorylation of the GluN1 subunit of the NMDA receptors and of Erk1/2^32,40^. These two pathways are directly involved in inflammatory pain processing. The P2RX4 channel is highly permeable to calcium ions^36^ and, in the hippocampus, activation of P2RX4 can only be observed following tetanic stimulations^19^. Inflammation-mediated hyper-excitability of sensory neurons might trigger the activation of pre-synaptic P2RX4 and allow sufficient calcium influx to release BDNF presumably from dense core vesicles. P2RX4 might therefore play a similar role to that of P2RX3, which contributes to pain processing by controlling the release of glutamate from sensory neuron pre-synaptic terminals^41^.

The link between P2RX4, BDNF and KCC2 is well documented in neuropathic pain models^42^. In these models P2RX4 is expressed by spinal microglia after peripheral nerve lesion and it controls the release of BDNF. Microglial BDNF in turn induces the down-regulation of KCC2, which causes local GABAergic disinhibition and hyper-excitability. Here we demonstrate that the potassium-chloride co-transporter KCC2 is also down-regulated in spinal cord L4-L6 dorsal horn, 24 hours after induction of peripheral inflammation. However, KCC2 downregulation seems independent of microglial activation or microglial BDNF expression, as we found little evidence for spinal microglial activation and little expression of BDNF in microglia. Therefore we can rule out any potential contribution of microglial BDNF to KCC2 down-regulation. BDNF is known to down-regulate KCC2 in rat inflammatory pain model^43^. BDNF released from sensory neurons is also involved in the establishment of CFA-evoked mechanical hypersensitivity^29,39,44^. Spinal infusion of the blocking TrkB-Fc antibody, blocks mechanical hypersensitivity evoked by paw injection of CFA^30^. Furthermore, specific deletion of the *bdnf* gene in Nav1.8 sensory neurons alleviates inflammatory pain without affecting neuropathic hypersensitivity^28^. Therefore neuronal BDNF is involved in inflammatory pain in a microglial-independent pathway. These observations are strengthened by the present study.

To summarize, our data indicate that P2RX4 expressed by sensory neurons controls BDNF-dependent KCC2 degradation in spinal cord neurons in conditions of long lasting inflammation. P2RX4 thus also lead to an increase of abnormal spinal hyper-excitability and consequently to the development of mechanical hyper-sensitivity.

## Materials and Methods

### Targeting of the P2RX4 gene and generation of mutant mice

Mice carrying a targeted null mutation of the P2RX4 gene were described elsewhere^19^. Briefly, a *E. Coli* ß-galactosidase (LacZ)-neomycin cassette was inserted in place of the first coding exon of the P2RX4 gene. In the resulting allele, the P2RX4 promoter drives ß-galactosidase expression. Chimeric mice were generated and crossed with C57BL/6 females to generate heterozygotes, which were then intercrossed to give rise to overtly healthy offspring in the expected Mendelian ratio. In the present study, mice were backcrossed for at least 20 generations and then maintained as separate P2RX4 knockout (P2RX4^−/−^) and wild-type (P2RX4^+/+^) lines. BDNF^HA/HA^ knock-in mice were obtained from Barbara Hempstead’s lab^26^.

Male mice, between 6 and 12 weeks of age, were maintained under a standard 12 h light/dark cycle with food and water available *ad libitum*. All experiments followed European Union (Council directive 86/609EEC) and institutional guidelines for laboratory animal care and use. Institutional license for hosting animals was approved by the French Ministry of Agriculture (No. D34-172-13).

### Induction of peripheral inflammation

Male mice received a single (30 µl) injection of Complete Freund’s Adjuvant (CFA, Sigma) in the plantar surface of the left hind paw. Inflammation was confirmed by the presence of edema and experiments were performed 24 h after injection.

### Complete Freund Adjuvant (CFA) induced mechanical hypersensitivity

CFA (30 µl) was injected in the hind paw of females P2RX4-KO and WT mice. Mechanical hyperalgesia was measured using an analgesimeter as described in^9^. Following the establishment of baseline thresholds, withdrawal thresholds were measured at 24 hours later.

### Primary culture of sensory neurons

Mice were euthanized with pentobarbital (300 mg/kg). L4, L5, L6 dorsal root ganglia were placed in cold PBS and stripped of axonal extension. Ganglia were enzymatically dissociated with type I collagenase (2 mg/mL, Gibco) and Dispase (5 mg/mL, Gibco) for 30 minutes at 37 °C and mechanically dissociated with a fire-polished Pasteur pipette. The cell suspension was centrifuged for 10 minutes at 800 g and re-suspended in MEMα medium (Gibco) supplemented with 10% horse serum (Life Technologies) and 1% penicillin-streptamycin (Gibco). Cells were then plated on poly-D-lysine (Sigma)-coated fluorodishes (WPI). Cells were maintained at 37 °C in a humidified atmosphere containing 5% CO_2_. Experiments were performed 24 h after plating.

### Primary and secondary antibodies

Primary antibodies used for immunohistochemistry were: Mouse anti-β-galactosidase (1:5000; ref 18637314, Promega), rabbit anti-TRPV1 (1:1000, ref sc-28759, Santa Cruz), rabbit anti-CGRP (1:1000, ref AB15360, Abcam), rabbit anti-substance P (1:500, ref 84500505, Biogenesis Ltd), rabbit anti-TrkA (1:500, ref SAB1305371, Sigma), goat anti-c-ret (1:500; ref AF482, R&D System), rabbit anti-phospho GluN1 (Ser896, 1:1000; Upstate), rabbit anti-phospho-Erk (1:1000, ref 9101, Cell Signalling), rabbit anti-HA (1:500, ref 715500, Life Technologies), mouse anti-Iba1 (1:2000, ref MNK4428, Wako), *Griffonia simplicifolia* isolectin (IB4) FITC conjugated (1:1000, Sigma). Secondary antibodies were Alexa Fluor 488 anti-rabbit or anti-goat antibody (1:2000) from Life Technologies, and Cy3-conjugated anti-mouse antibody (1:2000) was from Jackson ImmunoResearch Laboratories.

### Immunohistochemistry

Mice were euthanized by peritoneal injection of pentobarbital (300 mg/kg). Blood was removed with intracardiac perfusion of PBS. L4, L5, L6 DRG and lumbar spinal cords were placed for 2 h in 4% paraformaldehyde in phosphate buffer pH 7.4. For phospho-Erk and phosphor-GluN1 staining, a rapid intracardiac perfusion was delivered with a peristaltic pump at 20 ml/min, with 10 ml of Na_2_HPO_4_/NaH_2_PO_4_/NaCl buffer supplemented with 0.1 mM NaF, pH 7.4 followed by perfusion with 20 ml of 4% paraformaldehyde (PFA) in PBS 0.1 M, pH 7.5^45^. Tissues were cut with a vibratome into either 30 µm (spinal cord) or 20 µm (DRG) sections, rinsed with PBS and blocked with 10% goat serum diluted in a 0.1% Triton X100 solution, for 1 h. Appropriate primary antibody was added overnight at 4 °C. After rinsing, slices were incubated for 2 h with corresponding secondary antibody. Primary and secondary antibodies were diluted in PBS with 0.1% TritonX100. For phospho-staining, all buffers were supplemented with 0.1 mM NaF^45^. After rinsing, sections were mounted with Fluorescent Mounting medium (Dako) and observed either on a Zeiss AxioImager Apotome or on a Zeiss LSM780 confocal microscopes.

### Morphological analysis of neurons

Neuronal diameters of L4, L5, and L6 DRG slice sections were measured after immunohistochemistry. Neurons with distinguishable nuclei were counted and their area was measured using Metaview software. Soma diameters were also determined and three categories of sensory neurons were distinguished: *small* neurons with a diameter <17 µm, *intermediate* neurons diameter 17–25 µm, and *large* neurons >25 µm^46^. Five to eight sections from each DRG were analyzed and 3 to 5 animals were used for each condition. Experiments were performed three times.

### Quantification of microglial surface

Lumbar spinal cord slices were labelled with Iba1 antibody. 4 slices par animal and 3 mice per condition were analyzed. Iba1 positive area of the ipsilateral side of the CFA injection was detected after background subtraction with the ImageJ software and expressed in µm^2^.

### Retrograde labeling

30 µl of Fluorogold (Sigma, 4%) was injected into the glabrous skin of the left hind paw. Five days later, mice were sacrificed and L4, L5, L6 DRG were removed and fixed for 2 h in 4% paraformaldehyde before being sliced and labeled with the β-galactosidase antibody.

### Calcium imaging

The functional expression of membrane receptors was evaluated by recording the changes in the cytoplasmic Ca^2+^ concentration with a ratiometric fluorescent probe (Fura 2-AM, Life technologies, USA). Cells were loaded with 2 µM Fura 2-AM and 0.5% (w/v) BSA. After a 1 h incubation at room temperature, cells were washed three times with a saline solution containing (in mM): NaCl, 135; KCl, 5; CaCl_2_, 2; MgCl_2_, 2; glucose, 10; and Hepes, 10; pH 7.4. The Metafluor Imaging system (Universal Imaging) was used for fluorescence acquisition and analysis of individual cells. Fluorescence was excited by illumination with an x20 water immersion objective with a light wavelength switch provided by a DG4 filter wheel. Fluorescence was detected with a CCD camera under the Metafluor software control. Pairs of images were acquired every 2 s. Intracellular calcium is expressed throughout in the figures as the fluorescence ratio F_340_/F_380_, after background subtraction. Experiments were performed at room temperature.

Drugs were applied through an 8-channel gravity-fed bath-perfusion system, which allows switching between various solutions. ATP was prepared in distilled water and used at a final concentration of 20 µM. Ivermectin (Sigma) was dissolved in DMSO and used at a final concentration of 3 µM.

### Western blot

Ipsilateral L4, L5, L6 DRG were homogenized in lysis buffer (20 mM HEPES, pH 7.4, 100 mM NaCl, 5 mM EDTA, 1% Triton X-100, Complete Protease Inhibitor cocktail (Roche)), and ispi or contralateral dorsal horn of the spinal cord were homogenized in RIPA buffer (50 mM Tris-HCl pH 7.4, 150 mM NaCl, 1% Triton X-100, 0.5% Na-deoxycholate, and 0.1% SDS) containing Complete Protease Inhibitor cocktail. Lysates were clarified by centrifugation and protein concentration was determined using a protein assay kit (Biorad). Proteins were separated by reducing, 4–12%, SDS-PAGE, and transferred to a nitrocellulose membrane. The membrane was blocked with 5% non-fat dry milk/0.5% Tween 20 in Tris buffered saline (TBST) overnight at 4 °C. The membrane was incubated overnight at 4 °C with rabbit anti-P2RX4 (1:500, ref APR-002, Alomone Laboratories), KCC2 antibody (1:10000, ref 07–432, Millipore), mouse anti-tubulin (1:10000, ref T9026, Sigma) in TBST. After three washes in TBST, the membrane was then treated with HRP-conjugated secondary antibody for 45 min at room temperature and visualized with an ECL+ detection kit (Amersham). Uncropped western blot are presented in Sup. Fig. 5.

### Statistical analysis

All data are expressed as mean ± SEM. Data were analyzed with GraphPadPrism. Groups were compared using a Student’s t-test or two-way ANOVA. Cell counting and the distribution of the cell body size were analyzed with a χ^2^ analysis. The significance level was p < 0.05.

## Electronic supplementary material

Supplementary data
