# Supplementary material for: Sensory neuronal P2RX4 receptors controls BDNF signaling in inflammatory pain
Source: Sci Rep. 2018 Jan 17;8:964. doi: 10.1038/s41598-018-19301-5 (PMC5772667; doi:10.1038/s41598-018-19301-5)
Supplement: Supplementary file 1 — Supplementary data [file 41598_2018_19301_MOESM1_ESM.pdf]

Sensory neuronal P2RX4 receptors controls BDNF signaling in inflammatory pain.

Sarah Lalisse, Jennifer Hua, Manon Lenoir, Nathalie Linck, François Rassendren, Lauriane Ulmann

IGF, Labex ICST, CNRS, INSERM, Univ. Montpellier, 141 rue de la Cardonille, F-34094 Montpellier, France

Supplementary informations
5 supplemental figures + legends

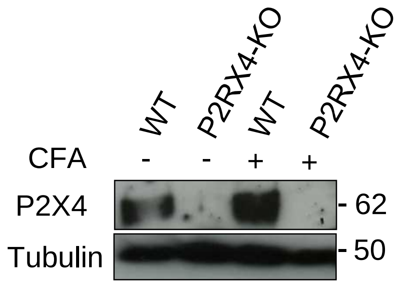

Sup figure 1: Representative Western blot of P2RX4 expression in DRG neurons central axons extracts. 24h following CFA injection in the hind paw, an increase of P2RX4 expression is seen in extracts of both ganglion and central processes, n=3 mice per lane.

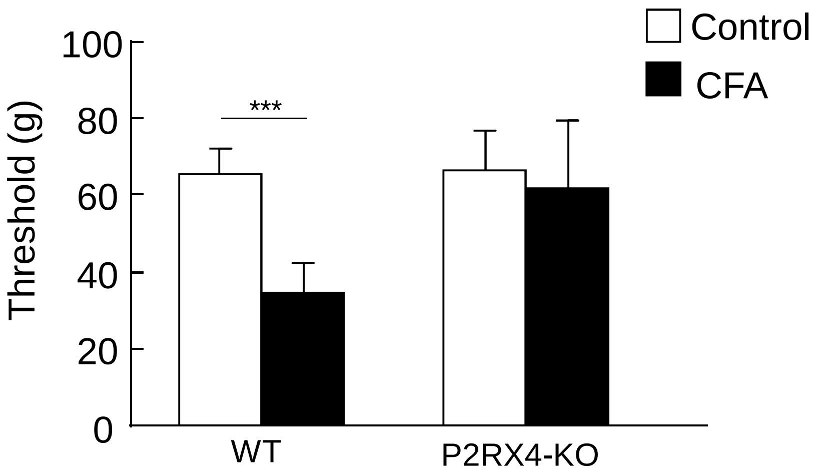

Sup figure 2: Lack of long lasting inflammatory pain in P2X4R-deficient female mice. Withdrawal threshold to mechanical stimulation was measured on injected side before and 24h after subcutaneous CFA injection in the paw. N=12 animals per group. Results are expressed as mean ± SEM, ***$p<0.005$, one-way ANOVA.

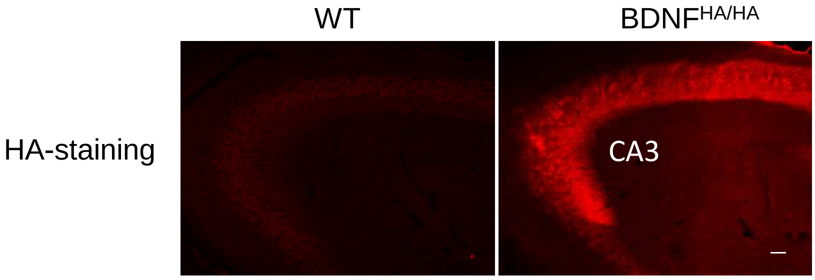

Sup figure 3: BDNF expression was analyzed in BDNF-HA mice in which an HA epitope is added at the carboxyl terminus of BDNF. **(A)** Demonstration of the specificity of the HA antibody in $BDNF^{HA/HA}$ mice. HA staining is clearly observed in the pyramidal cell layer of the CA3 area of the hippocampus of $BDNF^{HA/HA}$ mice (right panel) but not in WT animals (left panel). Scale bar 100 µm.

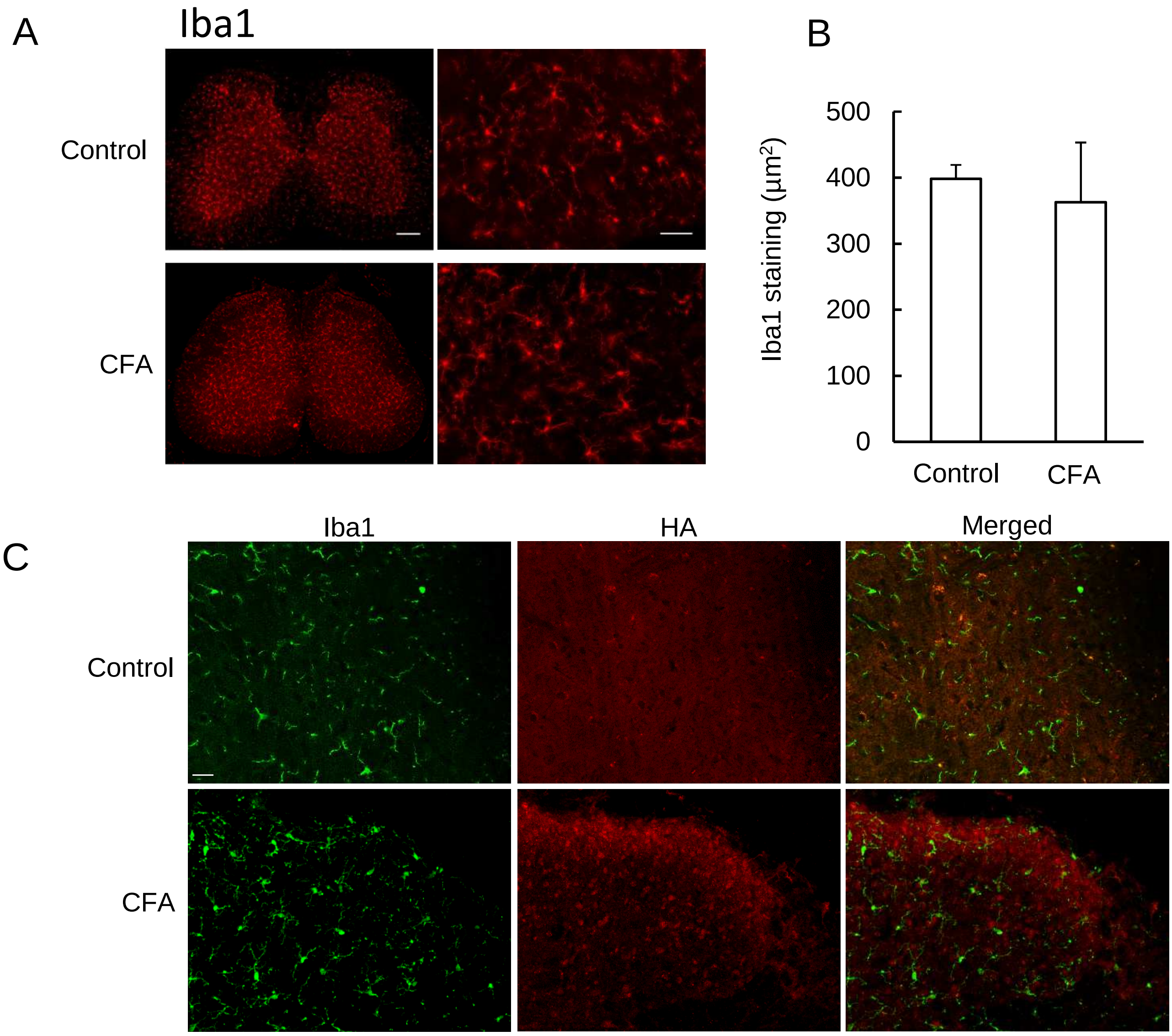

Sup figure 4: Absence of microglial activation in the spinal cord following induction of long lasting inflammation. (A) Microglial morphology analyzed by Iba1 staining 24h after CFA injection in WT and P2RX4-KO mice. CFA does not induce any detectable change in the morphology of microglia in the spinal cord, nor in the dorsal horn, ipsi lateral to the CFA injection. Scale bar 200 µm (left panel) and 100 µm (right panel). **(B)** Quantification of the Iba1 staining surface in the dorsal quadrant, ipsilateral to the injection side, indicates that CFA does not induce any significant change to microglial morphology. **(C)** CFA challenge does not induce BDNF expression in dorsal horn microglia. Experiments were performed in $BDNF^{HA/HA}$ mice. Double immunostaining performed with anti-Iba1 and anti-HA does not reveal any expression of BDNF in dorsal horn microglia 24h post CFA injection. Scale bar 20 µm.

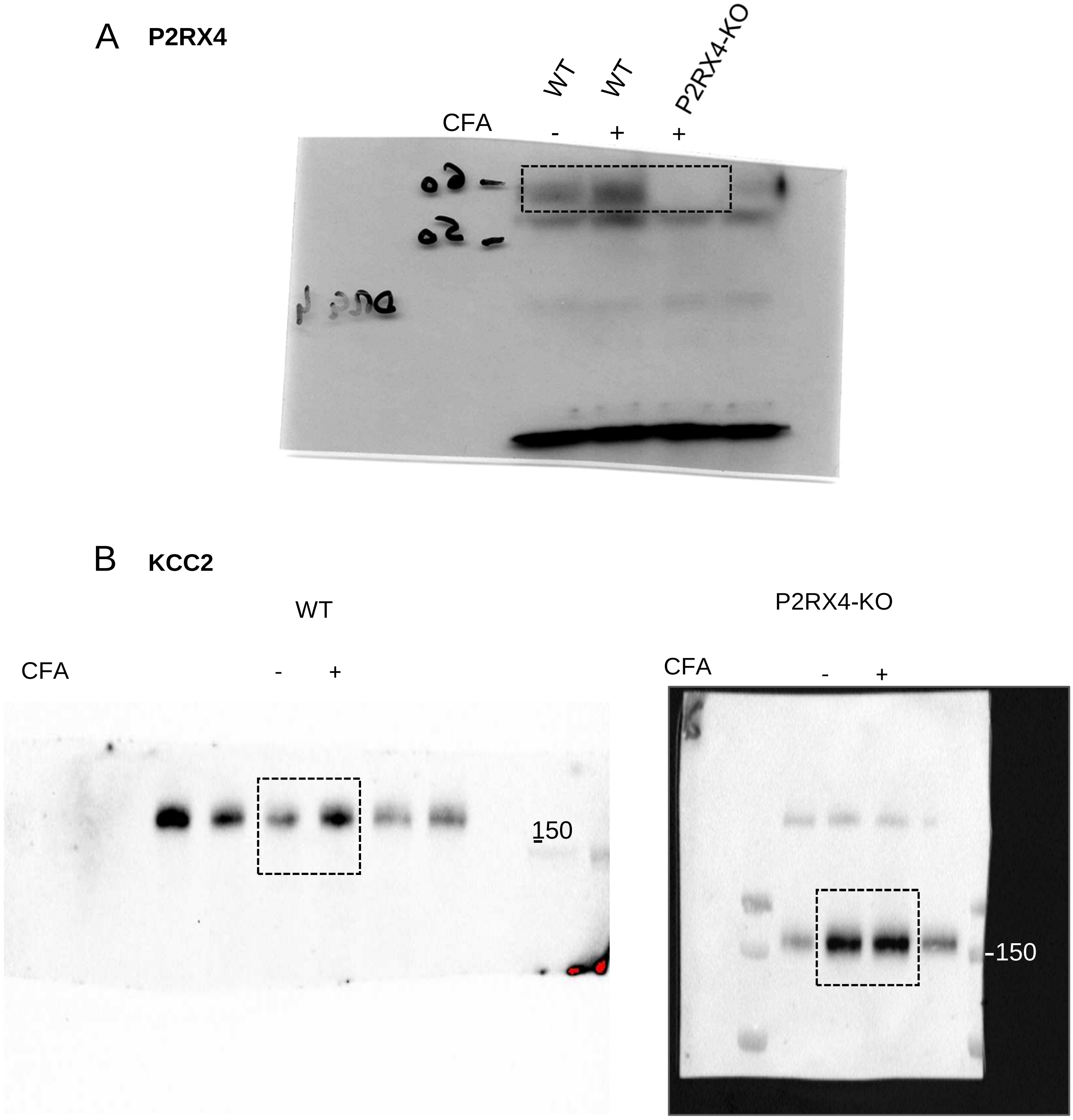

Sup figure 5 : Original uncropped images of western blot.

(A) Original uncropped images of P2RX4 western blot of DRG presented in fig. 3A. **(B)** Original uncropped images of KCC2 western blot of DRG presented in fig. 5. The dotted boxes indicate the cropped regions.
